# On a Complete Anchylosis of the Sacro-Iliac and Pubic Articulations, without Any Previous Morbid Condition

**Published:** 1842-10

**Authors:** 


ART. X.—G. VROLIK *ueber eine Vollkommene Verwachsung der Gelenke an den Kreuz-Darm-und Schaambeinen ohne vorhergegangene krankhafte Beschaffenheit.—Amsterdam*, 1841. Mit zwei Kupfertafeln. Fol., pp. 7.

*On a complete Anchylosis of the Sacro-Iliac and Pubic Articulations, without any previous morbid condition.* By G. VROLIK.—*Amsterdam.*

Two very rare cases are here mentioned. In one the symphysis pubis was completely anchylosed, with the exception of a small portion at its lower and posterior part, which was still filled up by ligament. The adjacent parts of the ossa innominata presented irregular surfaces, covered with short stalactitic osseous deposits, which sufficiently indicated that, in this case, the anchylosis was the result of long-continued inflammation of the bones and of the articulation between them.

In the other and more remarkable case these signs of previous disease are entirely absent. The situation of the symphysis pubis is occupied by healthy cancellous tissue with a small cavity in the centre; and the surfaces over both this symphysis and those between the sacrum and ilia are smooth and evenly-continuous with those of the adjacent bones. In short, there is no trace of the ordinary effects of diseases of bones or joints, except the osseous anchylosis of all the latter. In this respect the case is, we believe, unique; nor does it seem explicable except by regarding it as the result of defective original formation. For the natural anchylosis of certain joints, such as the cranial sutures and those between the portions of the sternum and sacrum, takes place according to a fixed rule, and therefore no such exceptional anchylosis as this can be referred to the same category with them. Neither can it be explained by any process of extra-uterine disease; for we know of none which produces osseous anchylosis without leaving many other signs of its effects. Congenital anchylosis, on the contrary, by whatever circumstances it may have been produced, seldom, if ever, presents any sign of disease of the adjacent parts.

The history of these cases is unfortunately unknown. The pelves are both from adult and, to all appearance, well-formed women.

